# Evaluation of myocardial injury induced by different ablation approaches (radiofrequency ablation versus cryoablation) in atrial flutter patients: a meta-analysis

**DOI:** 10.1042/BSR20182251

**Published:** 2019-05-24

**Authors:** Qing Zeng, XingSan Li, Ge Xu

**Affiliations:** Department of Cardiology, The First Affiliated Hospital of GuangXi Medical University, NanNing, China

**Keywords:** Atrial flutter, Cryoablation, myocardial injury, meta-analysis, pain perception, Radiofrequency

## Abstract

**Background:** To evaluate myocardial injury in Atrial flutter (AFL) patients undergoing Radiofrequency ablation (RF) and cryoablation (CRYO) treatments.

**Methods:** We conducted a systematic search on PubMed, Embase, Cochrane Library, and CBM databases. All relevant clinical trials (up to October 2018) on myocardial injury in AFL patients were retrieved and subsequent results analyzed with a random-effects model or a fixed-effects model.

**Results:** A total of eight clinical trials with a sample size of 644 patients, were identified and incorporated in the present study. The results indicated no significant differences in creatine kinase (CK) levels (mean difference (MD) = 62.74, *P*=0.46; 4–6 h and MD = 30.73, *P*=0.49; 12–24 h after ablation), creatine kinase MB(CK-MB) levels (MD = 17.32, *P*=0.25; 12–24 h post-ablation), troponinI (TnI) levels (MD = 0.12, *P*=0.08; 6 h after ablation), and troponin T (TnT) levels (MD = 0.30, *P*=0.08; 4–6 h post-ablation) between the two treatment approaches. However, patients receiving CRYO xhibited higher levels of CK (MD = 179.54, *P*=0.04; tested immediately after the procedure), CK-MB (MD = 10.08, *P*=0.004) 4–6 h after ablation, and TnT (MD = 0.19, *P*=0.002) tested the next morning. Moreover, those patients had a significantly reduced pain perception (odds ratio (OR) = 0.05, *P*=0.04) compared with those in the RF group.

**Conclusion:** These results indicate that CRYO in comparison with RF significantly increases myocardial injury in AFL patients. Additionally, it decreases pain perception during the procedure. Further large-sampled studies are needed to support these findings.

## Background

Atrial flutter (AFL) is a macroreentrant tachycardia propagating clockwise or counterclockwise through the cavotricuspid isthmus (CTI) and can cause stroke, heart failure, and significant subjective symptoms. To date, catheter ablation remains the recommended therapy for a wide variety of arrhythmias [1]. Ablation, whether radiofrequency ablation (RF) or cryoablation(CRYO) the two widespread ablation procedures, is the curative treatment for AFL [2,3].

Several studies have indicated an increase in myocardial injury biomarkers such as troponin T (TnT), [4,5] troponinI (TnI), [6–8] creatine kinase (CK), [9,10] and or its creatine kinase MB(CK-MB) after RF [10,11]. RF ablation can potentially cause serious complications [12,13]. CRYO of CTI, an alternative therapeutic approach, has been shown to reduce pain during the ablation procedure and decrease the risk of damage to the right coronary artery and the conduction system [14–17].

Our aim was to study and comparatively evaluate changes in levels of necrotic biomarkers, and the resulting pain perception during energy application.

## Methods

### Data sources and search strategy

The present meta-analysis was performed in accordance with the Preferred Reporting Items for Systematic Reviews and Meta-analyses (PRISMA) guidelines [18,19]. PubMed, Embase, Cochrane Library, and CBM databases were searched using the following keywords: Radiofrequency, Cryoablation, Atrial flutter, myocardial injury, and pain perception. No restrictions were imposed on language or date of publication. The final search was run on 1 October 2018. Additional searches were performed based on retrieved articles aiming to identify studies missed by prior searches.

### Study selection

**Figure 1:**
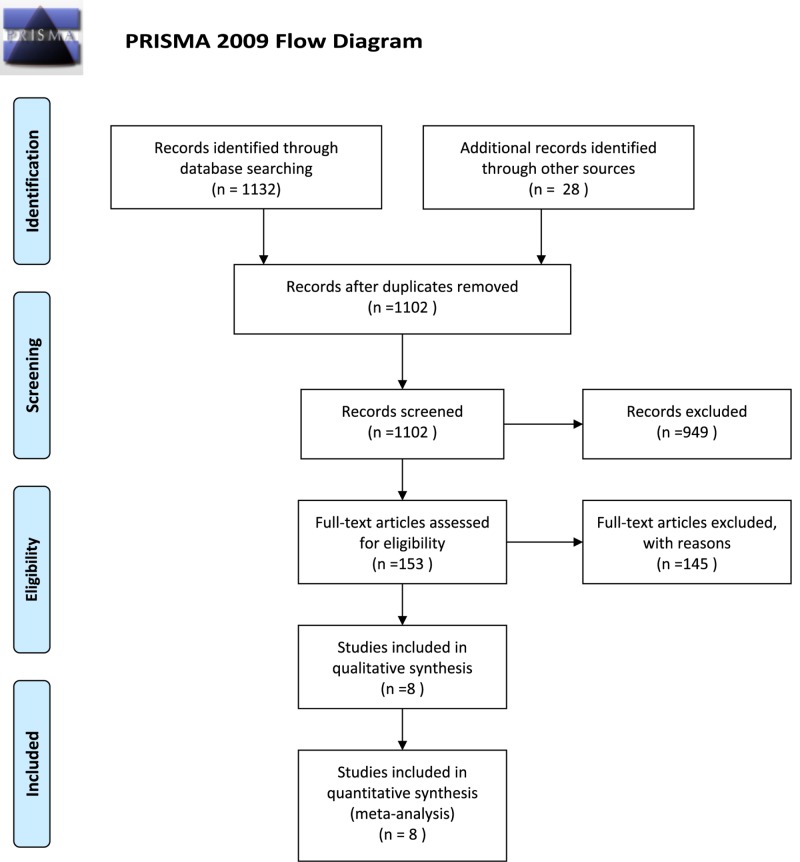
Selection process of studies included in the meta-analysis

All randomized controlled trials (RCTs) and quasi RCTs with a target population of AFL patients were included in the present study. The study selection diagram is shown in Figure 1. Randomized, crossover studies were also considered for inclusion.

RCTs with different outcomes than the ones of interest, studies lacking a comparable control or placebo, animal studies, reviews, meeting abstracts, and case-only studies were excluded.

### End point definition

End points of the present study are as follows: ‘CK levels (4–6 h and 12–24 h after ablation) (U/l)’, ‘CK-MB levels (4–6 h and 12–24 h after ablation) (U/l)’, ‘TnI levels (6 h post-ablation) (µg/l)’, ‘TnT levels (4–6 h after ablation and next morning) (µg/l)’, and ‘pain perception’.

### Data extraction and quality assessment

Data were extracted by two independent reviewers using a standardized data-extraction protocol and disagreements were resolved by consensus. Extracted data included: (i) study characteristics (title, first author name, year of publication, design, and duration); (ii) participant characteristics (age, sex, body mass index, presence of other chronic diseases such as hypertension, diabetes); (iii) outcome (CK levels, CK-MB levels, TnI levels, TnT levels, and pain perception). The quality of each RCT was evaluated using the Cochrane risk of bias instrument which, primarily assesses randomization and allocation concealment, the blinding process of individuals involved in the trial, the completeness of follow-up, and the outcome. Each study outcome was classified as ‘low risk of bias’, ‘unclear’, or ‘high risk of bias’.

### Data synthesis and statistical analysis

Statistical analysis was conducted by the Cochrane Review Manager (RevMan version 5.3). Continuous and dichotomous outcomes were analyzed using respectively mean differences (MDs) and pooled odds ratio (OR) to combine different tests and measurement scales within each domain. The overall effect estimates were calculated using inverse variance weighted fixed-effects analysis with a 95% confidence interval (CI). Standard deviations (SDs) were calculated using the following formula: SD = square root [(SD pretreatment)2 + (SD post-treatment)2 – (2R × SD pre-treatment × SD post-treatment)], assuming a correlation coefficient of (R) = 0.5.

Based on the Cochran Q statistic, heterogeneity among studies was identified using a standard χ^2^ test and a *P*-value (two-sided) [20]. *I^2^* index, as the percentage of variation across studies, was used to assess heterogeneity with *I^2^* values of 25, 50 or 75% representing low, moderate, or high heterogeneity, respectively [21]. The fixed-effect model was used for analysis when *I^2^* < 50% and the random-effect model when *I^2^* ≥ 50%. Subgroup analysis or sensitivity analysis methods were used to explore the sources of heterogeneity and to explain possible causes. We planned to construct a funnel plot for risk of publication bias evaluation given that the number of included studies was greater than 10.

## Results

### Study selection and characteristics

Of the initial 1160 studies identified by our primary search strategy, 58 duplicates were identified and removed. Further 949 studies were excluded after review of their titles and abstracts. Among the remaining 153 studies qualified for full-text review, 145 were excluded for the reason that 55 were published in the form of meta-analysis, abstracts, short communications or brief reports, 38 were animal studies, 24 were duplicated studies, and 28 did not report the outcomes of interest. Finally, six RCTs and two non-RCTs with a total sample size of 644 patients and an average follow-up period of 1day to 9 months were included in the present study (Figure 1 and Tables 1 and 2). The risk of bias among the included trials was generally low.

**Table 1:** Characteristics of eight clinical trials included in the meta-analysis

Study, year	Study design	Follow-up time	Outcome
Hernández-Romero, 2013 [22]	Non-random, Unblind, Control	2 months	CK, CK-MB, TnI
Oswald, 2007 [23]	Non-random, Unblind, Control	1 day	CK, CK-MB, TnT
Saygi, 2016 [24]	Single-blinded, Random, Control	1 day	TnI
Thornton, 2008 [16]	Random, Control	9 months	CK, CK-MB, TnT, Pain perception
Bastani, 2012 [14]	Single-blinded, Random, Control	6 months	Pain perception
Timmermans, 2003 [15]	Random, Control	6 months	Pain perception
Malmborg, 2009 [28]	Random, Control	6 months	Pain perception
Kuniss, 2009 [29]	Random, Control	3 months	Pain perception

**Table 2:** Characteristics of patients from eight clinical trials included in the meta-analysis

Study, year	Subjects (male)		Age (years)		Sex (male, %)		Hypertension (%)		Diabetes (%)		Body mass index (%)	
	Test Group	Control Group	Test Group	Control Group	Test Group	Control Group	Test Group	Control Group	Test Group	Control Group	Test Group	Control Group
Hernández-Romero, 2013 [22]	12	10	61.2	65.1	75	75	41.7	60	33.3	20	27.7	29.5
Oswald, 2007 [23]	10	9	62	68	80	100	37.5	37.5	37.5	37.5	30	27
Saygi, 2016 [24]	78	75	65	65	91	92	39	25	5	8	100	99
Thornton, 2008 [16]	32	30	55	56	84	93	NR	NR	NR	NR	NR	NR
Bastani, 2012 [14]	78	75	65	65	91	79	39.7	22.7	5.1	8	NR	NR
Timmermans, 2003 [15]	7	7	55	555	85.7	71.4	14.3	28.6	NR	NR	NR	NR
Malmborg, 2009 [28]	20	20	57	60	85	90	25	25	NR	NR	27.2	24.7
Kuniss, 2009 [29]	90	91	65	65	77.8	83.5	47.8	51.6	10	18.7	NR	NR

NR: Not described.

Detailed summary of involved studies and corresponding outcomes is as follows: four studies with CK levels as an outcome (three studies with a total of 22 patients in the CRYO group and 19 patients in the RF group and a timeline of 4–6 h post-ablation [16,22,23]; three studies with a total of 22 patients in the CRYO group and 19 patients in the RF group and a timeframe of 12–24 h [22,23,25]); three studies with CK-MB levels as an outcome of interest (three studies with a 4–6 h timeline, 22 patients in the CRYO group and 19 in RF group [16,22,23]; two studies with a 12–24 h timeline, 44 patients in the CRYO group and 40 in RF group [16,22]); two studies with TnI levels as an outcome (a time period of 6 h, a total of 90 patients enrolled in the CRYO group and 85 in the RF group [22,24]); two studies with TnT levels as an outcome of interest (a timeframe of 4–6 h and the next morning after ablation, 42 patients in the CRYO group and 39 patients in the RF group [16,23]) and finally, five studies with pain perception as outcome (CRYO, 227 patients; RF, 223 patients [14–16,28,29]).

### Risk of bias in included studies and quality of evidence

**Figure 2:**
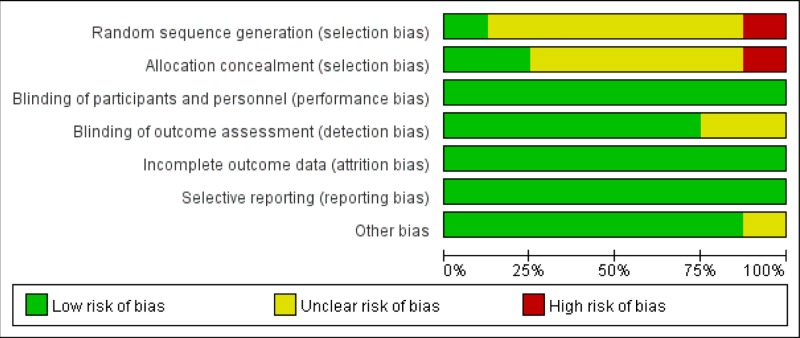
Risk of bias graph

**Figure 3:**
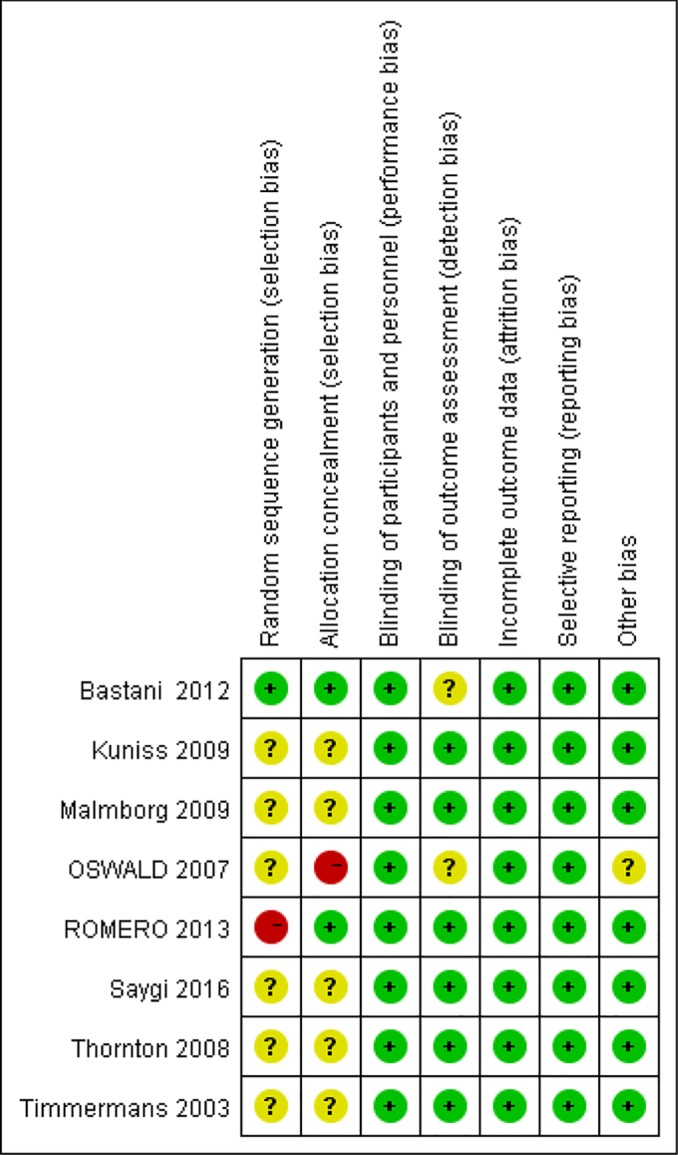
Risk of bias summary

The overall quality of the included studies, evaluated by the Cochrane risk of biases tool, was moderated and is shown in Figures 2 and 3. All studies were considered to have a low risk of bias in selective reporting according to the review of their protocols. All trials were regarded as having an unclear risk in other bias domain.

### Efficacy outcomes

#### Serum CK levels

**Figure 4:**
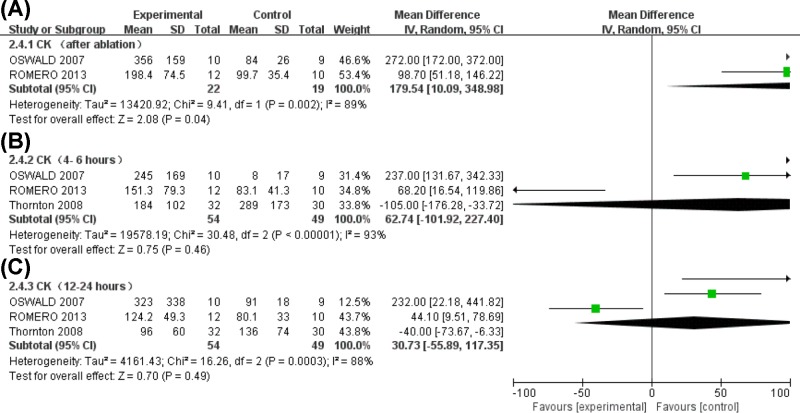
Forest plot for changes in serum CK levels

Substantial heterogeneity in serum CK levels was observed among studies with a significant increase seen in the CRYO group compared with the RF groups (MD = 179.54, 95%CI (95% confidence interval) (10.09, 348.98), *P*=0.04) minutes after the procedure (Figure 4A). Although not statistically significant, an increase in CK levels could be seen at 4–6 h (MD = 62.74, 95%CI (−101.92, 227.40), *P*=0.46) (Figure 4B) and 12–24 h (MD = 30.73, 95%CI (−55.89, 117.35), *P*=0.49) (Figure 4C) after ablation in the CRYO group.

**Figure 5:**
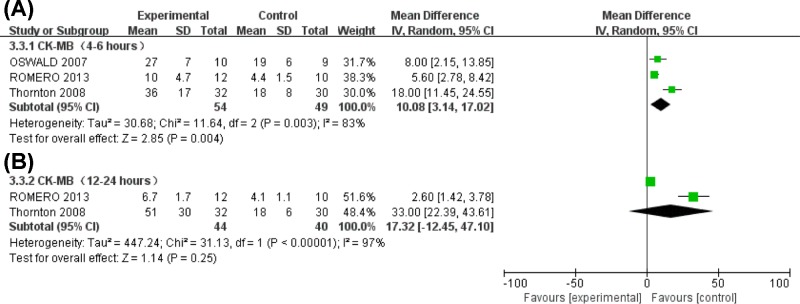
Forest plot for changes in serum CK-MB levels

**Figure 6:**
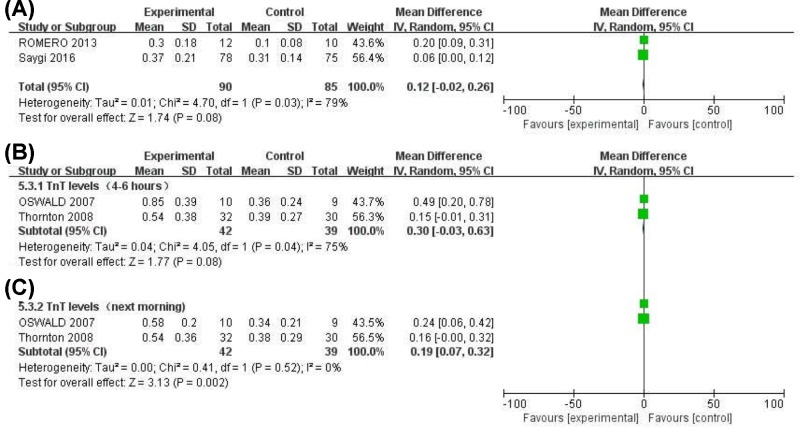
Forest plot for changes of serum TnI, TnT levels

**Figure 7:**
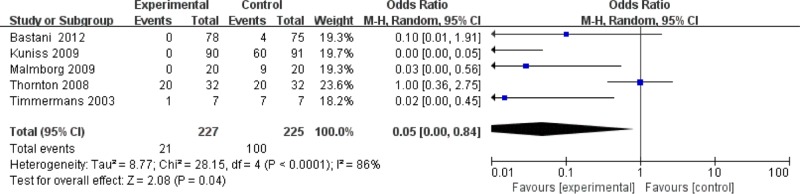
Forest plot for changes in pain perception

#### Serum CK-MB levels

Substantial heterogeneity in serum CK-MB levels was observed among studies 4–6 hours after ablation with a significant increase seen in the CRYO group compared with the RF group (MD = 10.08, 95%CI (3.14, 17.02), *P*=0.004] (Figure 5A). Additionally, a slight increase (MD = 17.32, 95%CI (−12.45, 47.10), *P*=0.25) (Figure 5B) in CK-MB levels can be seen 12–24 h after ablation in the CRYO group.

#### Serum TnI levels

The results indicated that CRYO treatment significantly elevated TnI levels (MD = 0.12, 95%CI (−0.02, 0.26), *P*=0.08) 6 h after ablation (Figure 6A).

#### Serum TnT levels

Substantial heterogeneity in serum TnT levels among studies was observed the next morning with a significant increase seen in the CRYO group as compared with RF group (MD = 0.19, 95%CI (0.07, 0.32), *P*=0.002] (Figure 6C). A slight increase in TnT levels was also observed 4–6 hours (MD = 0.30, 95%CI (−0.03, 0.63, *P*=0.08) (Figure 6B) after ablation in the CRYO group.

#### Pain perception

The results indicated that treatment with CRYO significantly reduced pain perception (OR = 0.05, 95%CI (0.00, 0.84), *P*=0.04) (Figure 7).

## Discussion

In RF cases, the injury usually results in much larger lesions [30,31] and seems to be associated with the site of ablation and the number of RF application [32]. Additionally, RF seems to favor the development of inflammatory infiltrates and fibrosis.

Ablation using cryothermal energy has several potential advantages including greater catheter stability due to better adherence to myocardial tissue, a lower risk of thrombus formation and systemic embolization, and a lower probability of myocardial perforation due to the preservation of tissue architecture [17,25–27,33].

This meta-analysis had several limitations. First, a susceptibility to bias due to smaller sample size and a limited number of clinical trials. Second, significant heterogeneity between studies could be observed. Additionally, biomarkers levels assessment started immediately after ablation.

There are also several concerns regarding clinical environment under which the cardiac necrosis biomarkers were used and then compared across different studies. In the work of Oswald et al. [23], patients with normal baseline values of cardiac biomarkers were included, while in the study of Thornton et al. [16] and Hernandez-Romero et al. [22], authors did not provide conditions under which patients were included in the study, as well as the basal level of their cardiac biomarkers. Therefore, it is difficult to assess and compare the effects of different studies since the clinical environment.

Therefore, the lack of baseline made it impossible to determine the confounding effect of baseline characteristics accurately. Third, there are risks of inaccurate conclusions due to potential heterogeneity among studies in terms of trial protocols, study populations, duration of ablation treatment. In summary, meta-analysis conclusions still need to be demonstrated by additional high-quality, large-sampled clinical studies.

## Conclusions

The present study indicates that CRYO significantly reduces pain perception and lowers discomfort during ablation. What was more, CRYO exhibited a higher occurrence of myocardial injury in comparison with RF.

## Declarations

All procedures performed in studies involving human participants were in accordance with the ethical standards of the institutional and/or national research committee and with the 1964 Helsinki declaration and its later amendments or comparable ethical standards. Informed consent was obtained from all individual participants included in the study.

## Abbreviations

AFL: atrial flutter
CK: creatine kinase
CK-MB: creatine kinase MB
CRYO: cryoablation
CTI: cavotricuspid isthmus
MD: mean difference
OR: odds ratio
RCT: randomized controlled trial
RF: radiofrequency ablation
SD: standard deviation
TnI: troponinI
TnT: troponin T
95%CI: 95% confidence interval

## References

[1] Hindricks G. (1993) Multicentre European Radiofrequency Survey (MERFS) Investigators of the Working Group on Arrhythmias of the European Society of Cardiology. The Multicentre European Radiofrequency Survey (MERFS): complications of radiofrequency catheter ablation of arrhythmias.. Eur. Heart J. 14, 1644–1653 10.1093/eurheartj/14.12.1644 8131762

[2] Blomström-Lundqvist C., Scheinman M.M., Aliot E.M. (2003) ACC/AHA/ESC guidelines for the management of patients with supraventricular arrhythmias—executive summary: a report of the American College of Cardiology/American Heart Association Task Force on Practice Guidelines and the European Society of Cardiology Committee for Practice Guidelines (Writing Committee to Develop Guidelines for the Management of Patients with Supraventricular Arrhythmias) developed in collaboration with NASPE-Heart Rhythm Society. J. Am. Coll. Cardiol. 42, 1493–1531 10.1016/j.jacc.2003.08.013 14563598

[3] Pérez F.J., Schubert C.M., Parvez B. (2009) Long-term outcomes after catheter ablation of cavo-tricuspid isthmus dependent atrial flutter: a meta-analysis. Circulation 2, 393–401 19808495 10.1161/CIRCEP.109.871665

[4] Hirose H., Kato K., Suzuki O. (2006) Diagnostic accuracy of cardiac markers for myocardial damage after radiofrequency catheter ablation. J. Interv. Card. Electrophysiol. 16, 169–174 10.1007/s10840-006-9034-4 17103317

[5] Katritsis D., Hossein-Nia M., Anastasakis A. (1997) Use of troponin-T concentration and kinase isoforms for quantitation of myocardial injury induced by radiofrequency catheter ablation. Eur. Heart J. 18, 1007–1013 10.1093/oxfordjournals.eurheartj.a015358 9183594

[6] Madrid A.H., del Rey J.M., Rubí J. (1998) Biochemical markers and cardiac troponin I release after radiofrequency catheter ablation: approach to size of necrosis. Am. Heart J. 136, 948–955 10.1016/S0002-8703(98)70148-6 9842005

[7] Manolis A.S., Vassilikos V., Maounis T. (1999) Detection of myocardial injury during radiofrequency catheter ablation by measuring serum cardiac troponin I levels: procedural correlates. J. Am. Coll. Cardiol. 34, 1099–1105 10.1016/S0735-1097(99)00330-7 10520797

[8] Brueckmann M., Wolpert C., Bertsch T. (2004) Markers of myocardial damage, tissue healing, and inflammation after radiofrequency catheter ablation of atrial tachyarrhythmias. J. Cardiovasc. Electrophysiol. 15, 686–691 10.1046/j.1540-8167.2004.03371.x 15175065

[9] Haines D.E., Whayne J.G., Walker J. (1995) The effect of radiofrequency catheter ablation on myocardial creatine kinase activity. J. Cardiovasc. Electrophysiol. 6, 79–88 10.1111/j.1540-8167.1995.tb00760.x 7780631

[10] Katritsis D.G., Hossein‐Nia M., Anastasakis A. (1998) Myocardial injury induced by radiofrequency and low energy ablation: a quantitative study of CK isoforms, CK‐MB, and troponin‐T concentrations. Pacing Clin. Electrophysiol. 21, 1410–1416 10.1111/j.1540-8159.1998.tb00212.x 9670185

[11] Bednarek J., Tomala I., Majewski J. (2004) Biochemical markers of myocardial damage after radiofrequency ablation. Kardiol. Pol. 60, 335–341 15226782

[12] de Ferrero L.A., Gil O.I. and Pedrote M.A. (2014) Spanish catheter ablation registry collaborators. Spanish catheter ablation registry. 13th official report of the Spanish society of cardiology working group on electrophysiology and arrhythmias (2013). Rev. Esp. Cardiol. (Engl. Ed.) 67, 925–35 25278211 10.1016/j.rec.2014.07.009

[13] Al Aloul B., Sigurdsson G., Adabag S. (2015) Atrial flutter ablation and risk of right coronary artery injury. J. Clin. Med. Res. 7, 270 10.14740/jocmr1986w 25699126 PMC4330022

[14] Bastani H., Drca N., Insulander P. (2012) Cryothermal vs. radiofrequency ablation as atrial flutter therapy: a randomized comparison. Europace 15, 420–428 10.1093/europace/eus261 22927662

[15] Timmermans C., Ayers G.M., Crijns H.J., Rodriguez L.M. (2003) Randomized study comparing radiofrequency ablation with cryoablation for the treatment of atrial flutter with emphasis on pain perception. Circulation 107, 1250–1252 10.1161/01.CIR.0000061915.06069.93 12628943

[16] Thornton A.S., Janse P., Alings M. (2008) Acute success and short-term follow-up of catheter ablation of isthmus-dependent atrial flutter; a comparison of 8 mm tip radiofrequency and cryothermy catheters. J. Interv. Card. Electrophysiol. 21, 241–248 10.1007/s10840-008-9209-2 18363087 PMC2292475

[17] Collins N.J., Barlow M., Varghese P. (2006) Cryoablation versus radiofrequency ablation in the treatment of atrial flutter trial (CRAAFT). J. Interv. Card. Electrophysiol. 16, 1–5 10.1007/s10840-006-9027-3 17024571

[18] Moher D., Liberati A., Tetzlaff J. (2009) PRISMA GroupPreferred reporting items for systematic reviews and meta-analyses: the PRISMA statement. PLoS Med. 6, e1000097 10.1371/journal.pmed.1000097 19621072 PMC2707599

[19] Liberati A., Altman D.G., Tetzlaff J. (2009) The PRISMA statement for reporting systematic reviews and meta-analyses of studies that evaluate health care interventions: explanation and elaboration. PLoS Med. 6, e1000100 10.1371/journal.pmed.1000100 19621070 PMC2707010

[20] Higgins J., Thompson S., Deeks J. (2002) Statistical heterogeneity in systematic reviews of clinical trials: a critical appraisal of guidelines and practice. J. Health Serv. Res. Policy 7, 51–61 11822262 10.1258/1355819021927674

[21] Hase M., Babazono T., Ujihara N. (2013) Comparison of spironolactone and trichlormethiazide as add‐on therapy to renin–angiotensin blockade for reduction of albuminuria in diabetic patients. J. Diabetes Invest. 4, 316–319 10.1111/jdi.12029 24843672 PMC4015670

[22] Hernández‐Romero D., Marin F., Roldan V. (2013) Comparative determination and monitoring of biomarkers of necrosis and myocardial remodeling between radiofrequency ablation and cryoablation. Pacing Clin. Electrophysiol. 36, 31–36 10.1111/pace.12017 23078110

[23] Oswald H., Gardiwal A., Lissel C. (2007) Difference in humoral biomarkers for myocardial injury and inflammation in radiofrequency ablation versus cryoablation. Pacing Clin. Electrophysiol. 30, 885–890 10.1111/j.1540-8159.2007.00776.x 17584270

[24] Saygi S., Drca N., Insulander P. (2016) Myocardial injury during radiofrequency and cryoablation of typical atrial flutter. J. Interv. Card. Electrophysiol. 46, 177–181 10.1007/s10840-015-0074-5 26546105

[25] Montenero A.S., Bruno N., Antonelli A. (2005) Comparison between a 7 French 6 mm tip cryothermal catheter and a 9 French 8 mm tip cryothermal catheter for cryoablation treatment of common atrial flutter. J. Interv. Card. Electrophysiol. 13, 59–69 10.1007/s10840-005-0353-7 15976981

[26] Feld G.K., Daubert J.P., Weiss R. (2008) Cryoablation Atrial Flutter Efficacy (CAFÉ) Trial Investigators. Acute and long-term efficacy and safety of catheter cryoablation of the cavotricuspid isthmus for treatment of type 1 atrial flutter. Heart Rhythm 5, 1009–14 10.1016/j.hrthm.2008.03.019 18598956

[27] Wadhwa M.K., Rahme M.M., Dobak J., Li H., Wolf P., Chen P. (2000) Transcatheter cryoablation of ventricular myocardium in dogs. J. Int. Card Electrophysiol 4, 537–46 10.1023/A:1009872917450 11046193

[28] Malmborg H., Lönnerholm S. and Lundqvist C.B. (2009) A prospective randomised comparison of large-tip cryoablation and 8-mm-tip radiofrequency catheter ablation of atrial flutter. J. Interv. Card. Electrophysiol. 24, 127–131 10.1007/s10840-008-9315-1 18987965

[29] Kuniss M., Vogtmann T., Ventura R. (2009) Prospective randomized comparison of durability of bidirectional conduction block in the cavotricuspid isthmus in patients after ablation of common atrial flutter using cryothermy and radiofrequency energy: the CRYOTIP study. Heart Rhythm 6, 1699–1705 10.1016/j.hrthm.2009.09.012 19959115

[30] Grubman E., Pavri B.B., Lyle S. (1999) Histopathologic effects of radiofrequency catheter ablation in previously infarcted human myocardium. J. Cardiovasc. Electrophysiol. 10, 336–342 10.1111/j.1540-8167.1999.tb00680.x 10210495

[31] Carlsson J., Erdogan A., Guettler N. (2001) Myocardial injury during radiofrequency catheter ablation: comparison of focal and linear lesions. Pacing Clin. Electrophysiol. 24, 962–968 10.1046/j.1460-9592.2001.00962.x 11449593

[32] Kimman G.P., Theuns D., Szili-Torok T. (2004) CRAVT: a prospective, randomized study comparing transvenous cryothermal and radiofrequency ablation in atrioventricular nodal re-entrant tachycardia. Eur. Heart J. 25, 2232–2237 10.1016/j.ehj.2004.07.008 15589641

[33] Hanninen M., Yeung‐Lai‐Wah N., Massel D. (2013) Cryoablation versus RF ablation for AVNRT: a meta‐analysis and systematic review. J. Cardiovasc. Electrophysiol. 24, 1354–1360 10.1111/jce.12247 24016223

